# Observation of a black‐cheeked waxbill (*Brunhilda charmosyna*) cleaning a Kirk’s dik‐dik (*Madoqua kirkii*)

**DOI:** 10.1002/ece3.8506

**Published:** 2022-02-14

**Authors:** Brendah Nyaguthii, Peter Njoroge, Damien R. Farine

**Affiliations:** ^1^ Department of Ornithology National Museums of Kenya Nairobi Kenya; ^2^ Department of Wildlife School of Natural Resource Management University of Eldoret Eldoret Kenya; ^3^ Mpala Research Centre Nanyuki Kenya; ^4^ Department of Evolutionary Biology and Environmental Studies University of Zurich Zürich Switzerland; ^5^ Department of Collective Behavior Max Planck Institute of Animal Behavior Konstanz Germany

**Keywords:** cleaning mutualism, commensalism, interspecific interactions

## Abstract

The vast majority of interspecific interactions are competitive or exploitative. Yet, some positive interspecies interactions exist, where one (commensalism) or both (mutualism) species benefit. One such interaction is cleaning mutualisms, whereby a cleaner removes parasites from a client. In this note, we document the novel observation of a black‐cheeked waxbill (*Brunhilda charmosyna*) appearing to clean a Kirk's dik‐dik (*Madoqua kirkii*), at the Mpala Research Centre in Laikipia County, Kenya. The purported cleaning took place for over one minute and is notable firstly for the dik‐dik remaining still for the duration of cleaning and secondly for involving two species that are much smaller than those traditionally involved in bird–mammal cleaning interactions. Unfortunately, no further cleaning events were subsequently observed, raising questions about whether this record was opportunistic or a regular occurrence. Future observations may reveal whether this behavior is widespread and whether it involves other small passerines.

## Graphical abstract

A novel observation of a cleaning interaction between a black‐cheeked waxbill (*Brunhilda charmosyna*) and a Kirk's dik‐dik (*Madoqua kirkii*), in Laikipia County, Kenya.

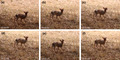

## 1 INTRODUCTION

Positive–positive (mutualistic) or positive–neutral (commensalistic) interactions between species have long fascinated naturalists (Boucher et al., 1982). Among vertebrates, mutualisms have been described between foragers, including humans communicating with greater honeyguides (*Indicator indicator*) to find bee's nests (Spottiswoode et al., 2016) and in mixed‐species foraging flocks of birds and mammals that share vigilance for predators (Hino, 1998). One type of mutualism that has received a substantial amount of attention is cleaning mutualisms. Cleaning mutualisms are a specific type of interspecies interaction where one species feeds on anything that is affecting the other in a negative way, usually parasites. They range in importance for the species involved, from obligate cleaners who get the majority of their diet from cleaning, to facultative cleaners that engage in cleaning interactions more opportunistically (Vaughan et al., 2017).

Cleaning mutualisms are very common in coral reef communities (Caves, 2021). They have also been recorded among mammals, including bats that forage around ungulates and reduce the number of biting flies (Palmer et al., 2019), northern raccoons (*Procyon lotor*) that groom the heads of key deer (*Odocoileus virginianus clavium*) (Cove et al., 2017), and coatis (*Nasua narica*) that groom tick off the Baird's tapir (*Tapirus bairdii*) (McClearn, 1992). There are also some well‐known bird‐ungulate examples, including oxpeckers (*Buphagus* spp.) that perch on—and collect parasites from—large herbivores (Dean & Macdonald, 1981). However, while cleaner interactions in reef communities, and among some mammals, are relatively active choices by both participants (e.g., clients coming to cleaning stations and posing by remaining still and, in some cases, exposing parts of their body), bird–mammal relationships appear more passive on the part of the client (Sazima, 2011), and many are arguably commensalistic at best (McElligott et al., 2004), with birds often taking advantage of large mammals. Further, most cases of cleaning involve relatively large birds and ungulates (Sazima, 2011), with few reports coming from smaller species. Smaller “cleaner” species are generally linked to more parasitic relationships with hosts, such as sharp‐billed ground finches (*Geospiza difficilis*) that probe wounds on seabird hosts for blood (Koster & Koster, 1983). Thus, large size differentials between species may be more likely to be linked to a positive–negative relationship between the “cleaner” and the “client.”

Here, we describe an observation of a small bird—the black‐cheeked waxbill (*Brunhilda charmosyna*)—seemingly cleaning a posing male Kirk's dik‐dik (*Madoqua kirkii*). The observation took place over several minutes at close range in open habitat. These two species are among the smallest to be recorded as being involved in a bird–mammal cleaning interaction, and the observation provides a rare case in which a mammalian client poses for a small bird.

## 2 METHODS

The observation took place within the Mpala Research Centre in Kenya. The Mpala Ranch is a 48,000‐hectare property in the center of Laikipia County and was established as a scientific research station in 1994. Within the Mpala Ranch is the research accommodation (the Mpala Research Centre) where DRF and BN were staying at the time the observation was made. The observation started on the 27th of March 2021 at approximately 14:31:00, as DRF walked behind his banda and noticed a Kirk's dik‐dik with a black‐cheeked waxbill perched on its horns. After observing for some seconds, DRF then took a series of images, from 14:32:00 to 14:32:14 using the built‐in camera in a Fairphone 3. The observation ended shortly after the final image was taken.

## 3 RESULTS

**FIGURE 1:**
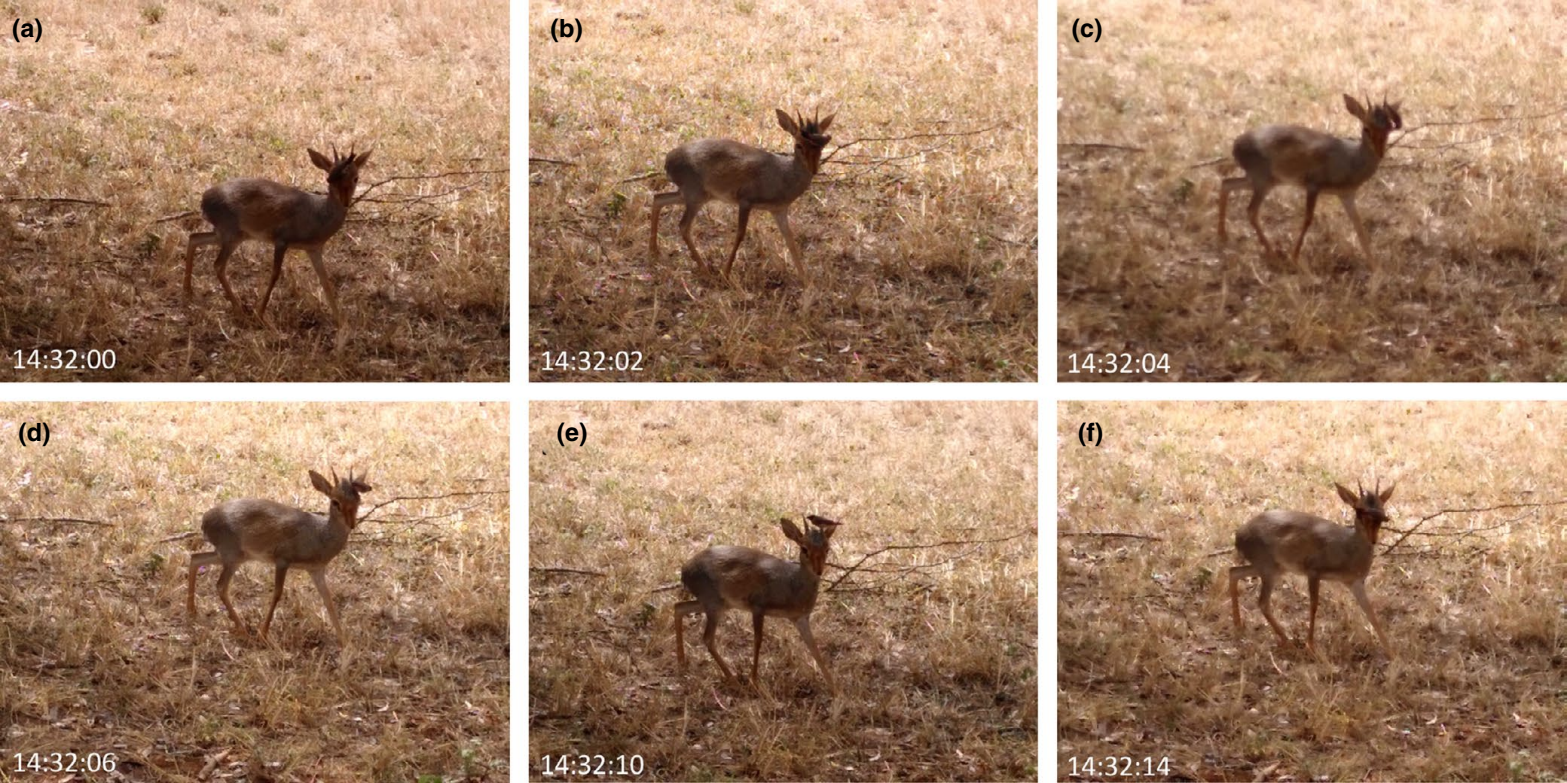
Sequence of images captured involving a black‐cheeked waxbill appearing to clean a Kirk's dik‐dik. (a) In the first image (timestamp on the bottom‐left), the black‐cheeked waxbill is probing the right eye, before (b) moving to (c) the left eye, then (d) the left ear, back to (e) the horns (where it was when the observation initially started), and finally back to (f) the left eye. The interaction ended shortly after the last image was captured

The observation lasted at least 1 min, but the interaction was already underway when first noticed. For the duration of the interaction, the Kirk's dik‐dik remained immobile, while the black‐cheeked waxbill moved around its head, probing its horns (especially at the base), both ears, both eyes, and around the snout (Figure 1). The black‐cheeked waxbill moved repeatedly between different areas of the head of the Kirk's dik‐dik, all the while prompting no clear movement response by the dik‐dik. The interaction ended when the black‐cheeked waxbill was probing the left eye, appearing to prompt the Kirk's dik‐dik to shake it from its head.

## 4 DISCUSSION

We describe what is, to our knowledge, the first possible case of a cleaning interaction involving either an estrildid finch (family *Estrildidae*) or a dik‐dik (*Madoqua* spp.). This observation is notable for two reasons. First, by remaining still during the entire interaction, the Kirk's dik‐dik appeared to be posing for cleaning by the black‐cheeked waxbill. Second, it involved two of the smallest species known to participate in a bird–mammal cleaning interaction. This observation, especially if it can be reinforced by future similar observations, might help to shed light on the drivers of variation in behavior among bird–mammal cleaning interactions, such as the role of size differences in determining whether the relationship between species is active (i.e., where the client poses) or passive (i.e., when the client is unconcerned). Further, increasing the taxonomic breadth of descriptions of facultative cleaners could help give insights into the first evolutionary steps toward more dedicated/obligate cleaning systems.

The defining feature of classical cleaning mutualisms, such as those on coral reefs, is the transaction that takes place between cleaners and clients. Clients seek out cleaning stations, with cleaners making economic decisions about which clients to service (Bshary & Noë, 2003), while clients can also choose the highest value cleaners (Bshary & Schäffer, 2002). By contrast, most bird–mammal cleaning interactions are passive, with “clients” not seeking cleaners and remaining relatively unconcerned to the presence of cleaners (Sazima, 2011). For example, eastern phoebes (*Sayornis phoebe*) will occasionally take parasites directly from clients (in this case, white‐tailed deer (*Odocoileus virginianus*), but mostly catch insects that the client has disturbed from the vegetation and will not prompt any response by the client (Baruzzi et al., 2017). Some mammalian clients do facilitate cleaning, by remaining still or even presenting specific parts of their body to allow themselves to be “serviced” (Sazima, 2011). For example, capybaras (*Hydrochoerus hydrochaeris*) (Tomazzoni et al., 2005) remain still and present specific body parts for cleaning. Cursory examination of the review on cleaning interactions in birds by Sazima (2011) points to relative body size as a potentially important factor in determining client behavior, suggesting that body size differences may predispose relationships to be more or less active from the perspective of the client. Large domestic cattle and megaherbivores pay little attention to cleaners, whereas smaller mammals or those cleaned by larger avian cleaners appear to be more prone to posing (Sazima, 2011). This prediction matches our observation, in which one of the smallest species of ungulates clearly posed while being serviced by a small bird which a larger ungulate species might have ignored.

Among smaller bird species, their relationships with ungulates are likely to generally be more commensalistic or even parasitic. For example, many birds follow herds of ungulates, flycatching insects that are flushed by the mammals. This may bring minor benefits to the mammals—as evidenced by bats reducing the abundance of biting flies (Palmer et al., 2019)—at no additional cost, but is unlikely to represent a strong mutualism (where both species benefit equally). Many other interactions with small birds may even be costly for their larger “clients,” such as Galapagos mocking birds (*Nesomimus* spp.) and oxpeckers (*Buphagus* spp.) that target wounds on masked boobies (*Sula dactylatra*) (Curry & Anderson, 1987) and large ungulates (Weeks, 2000), respectively. However, we expect that the purported cleaning interaction between the black‐cheeked waxbill and the Kirk's dik‐dik was positive. Black‐cheeked waxbills have a diet predominately consisting of small seeds and herbage, with insects and nectar also taken opportunistically (Skead, 1976) and if targeting wounds this who would not have prompted the Kirk's dik‐dik to remain immobile during the interaction. The waxbill's diet also raises the question of what exactly it was feeding on while perching on the dik‐dik. While the granivorous diet of waxbills means that it could have been seeking seeds stuck in the fur, it could also have been gleaning for ectoparasites. The latter could explain why the waxbill's probing was focused around the head of the dik‐dik, as censuses of the distribution of parasites such as ticks on mammal bodies have found them to be predominately around the head and neck of individuals (Bittencourt & Rocha, 2002). However, this would not explain why the waxbill appeared to specifically glean in the ears and around the eyes of the dik‐dik, which could also suggest that it may have been targeting secretions.

We hope that this record will stimulate closer observations across the range of the four dik‐dik species, all of which are likely to overlap regularly with a range of small birds, including waxbills. As facultative cleaners express their behaviors more rarely, they are also more difficult to observe and study, meaning that they could be underdescribed. A key question that emerges is whether such a behavior—especially in facultative cleaners—might be learnt, as recently suggested in coral reef cleaning mutualisms (Truskanov et al., 2020). Learning would allow innovations by some individuals to spread to new populations. Given that black‐cheeked waxbills appear at least somewhat nomadic—their abundance at the Mpala Research Centre varies considerably over time (BN, DRF, *pers*. *obs*.)—this could allow the behavior to spread widely. However, if at the same time, the behavior requires learning on the part of the client, then local innovations involving a few cleaners and clients may well rapidly go extinct if one participant, in this case the cleaner, leaves and does not come back to the same location. Such conditions (a mobile cleaner and a resident client) might limit the spread of cleaning innovations relative to the opposite (a resident cleaner and a mobile client). In fact, one observed feature of many obligate cleaning interactions is that cleaners have relatively stable location to facilitate repeated interactions with clients (Hobson, 1971). The rarity of cleaning between waxbills and dik‐diks (currently just this single record) could therefore be because, in this case, the clients are the ones with the small range while the cleaners move widely across the landscape.

## CONFLICT OF INTEREST

The authors declare no conflicts of interest.

## AUTHOR CONTRIBUTIONS

**Brendah Nyaguthii:** Conceptualization (equal); Methodology (equal); Writing – original draft (equal); Writing – review & editing (equal). **Peter Njoroge:** Conceptualization (equal); Writing – original draft (equal); Writing – review & editing (equal). **Damien Farine:** Conceptualization (equal); Data curation (equal); Funding acquisition (lead); Investigation (equal); Methodology (equal); Writing – original draft (equal); Writing – review & editing (equal).

## Data Availability

There are no data associated with this submission (all images are presented in Figure 1).
